# Combination of Intranasal insulin and oral semaglutide for cognition in older adults with metabolic syndrome at high dementia risk

**DOI:** 10.1002/alz70860_102127

**Published:** 2025-12-23

**Authors:** Hanadi Shamrok, Tal davidy, Iscka Yore, Tal Niv, Tali C Yaffe, Ramit Ravona‐Springer, Abigail Livny, Orit H. Lesman‐Segev, Owen T. Carmichael, Dimitrios Kapogiannis, Mary Sano, Michal S Beeri

**Affiliations:** ^1^ The Joseph Sagol Neuroscience Center, Sheba Medical Center, Ramat‐gan, Israel; ^2^ sheba medical center, Ramat gan, Israel; ^3^ The Joseph Sagol Neuroscience Center, Sheba Medical Center, Tel Hashomer, Israel; ^4^ Sheba Medical Center, Ramat Gan, Israel; ^5^ Memory clinic and The Joseph Sagol Neuroscience Center, Sheba Medical Center, Israel, Ramat‐Gan, Israel; ^6^ Pennington Biomedical Research Center, Baton Rouge, LA, USA; ^7^ National Institute on Aging, Baltimore, MD, USA; ^8^ Icahn School of Medicine at Mount Sinai, New York, NY, USA; ^9^ Herbert and Jackeline Krieger Klein Alzheimer's Research Center, Rutgers Biomedical and Health Sciences, newark, NJ, USA

## Abstract

**Background:**

We have initiated a randomized double‐blind placebo‐controlled feasibility trial combining intranasal insulin (INI) with semaglutide, a GLP‐1 receptor agonist, to improve cognition in older adults with metabolic syndrome (MetS) and mild cognitive impairment (MCI). Since both INI and semaglutide have beneficial effects on cerebrovascular disease and on insulin signaling, we anticipate their improvements will underlie the hypothesized cognitive benefits.

**Method:**

This trial will include 80 older adults aged > 60 years with MetS and MCI, randomized to one of four 12‐month self‐administered treatments: INI/oral semaglutide, intranasal placebo/oral semaglutide, INI/oral placebo, and intranasal placebo/oral placebo. Feasibility of combining INI with semaglutide will be tested by examining the ease of use of INI (20IU, twice/day) with semaglutide (14g, once/day), adherence, and safety profile. Exploratory outcomes of efficacy include global cognition and neurobiological markers: cerebral blood flow, cerebral glucose utilization, white matter hyperintensities, Alzheimer's related blood biomarkers (*p*‐tau 217, neurofilament light chain, and glial fibrillary acidic protein) and expression of insulin signaling proteins measured in brain‐derived exosomes. Feasibility and efficacy will be assessed in the intent‐to‐treat sample.

**Result:**

Data collection began in March 2024. So far 562 people completed a pre‐screening online questionnaire, with 190 passing pre‐screening of whom 54% met eligibility criteria. Of the 73 participants who were screened face‐to‐face so far, 33% (*N* = 24) were eligible. Of those deemed ineligible, 44% did not meet criteria for MCI, 44% did not meet criteria of MetS, and 12% did not want to participate. Nineteen participants were randomized, 2 dropped out before beginning treatment and 5 are awaiting randomization. Randomized participants have an average age of 66.7 years [SD=5.26], BMI of 32.8 [SD=5.7], MMSE of 25.0 [SD=2.6] and Hba1c of 5.6 [SD=0.31]; 61% are women.

**Conclusion:**

This feasibility study is anticipated to provide the basis for a multi‐center large‐scale randomized clinical trial investigating the cognitive benefits of the combination of INI and oral semaglutide in individuals with MetS and at high dementia risk. This combination targets multiple pathways implicated in cognitive decline, potentially offering a novel therapeutic approach for the understudied yet highly prevalent and high‐risk MetS population.

